# A dual-channel secondary closed-loop supply chain considering retail groups and fairness concerns

**DOI:** 10.1371/journal.pone.0292753

**Published:** 2023-10-16

**Authors:** Yue Tan, Chunxiang Guo

**Affiliations:** Business School, Sichuan University, Chengdu, P.R. China; University of Victoria / Universiti Teknologi Malaysia /, CANADA

## Abstract

The rise of retail groups has strengthened their voice in the supply chain, drawing more attention from supply chain members to the issue of profit fairness. To explore the influence of fairness concerns on operational decisions in closed-loop supply chains after the formation of retail groups. In this paper, we first construct a secondary dual-channel closed-loop supply chain led by a retail group and followed by a manufacturer. Next, the corresponding game models are constructed under three scenarios, namely, fairness neutrality (FN), fairness concerns of the retail groups (FR), and fairness concerns of the manufacturer (FM), respectively. Finally, the game models are solved and analyzed. It turns out that it is easier for the manufacturer to satisfy its demands for fairness by adjusting the wholesale price. Furthermore, we find that fairness concerns do not enhance the recycling rate of used products and the greenness of remanufacturing. For retail groups, fairness concerns can hurt their profits, but appropriate fairness concerns can contribute to profitable growth in their retail business. Interestingly, the manufacturer’s fairness concerns do not affect the total profitability of the supply chain system, but the retail group’s fairness concerns do. This paper identifies dual changes in the scope of operations and power structure of retail groups in closed-loop supply chains, as well as analyzes the fairness concerns raised by these changes, which will lead to new recommendations for operational decision-making in firms.

## 1. Introduction

Environmental pollution and resource scarcity have become common global challenges. People produce 2.01 billion tons of solid waste yearly, and it will increase 69.15% by 2050 [1]. The closed-loop supply chain(CLSC) is an effective way to enhance resource utilization and reduce environmental pollution [2–4]. Remanufactured products cost 40–65% less than new products and require 85% less energy [5]. Therefore, companies are actively participating in closed-loop supply chains for sustainable development [6]. As the critical link between the market and the business, more and more retailers are getting their act together [7]. They opened up dual channel sales while expanding their recycling business, eventually becoming a retail group. This is positive for the formation of a closed-loop supply chain [8, 9].

For example, Amazon, the largest online retailer in the United States, has also opened physical stores, such as Amazon Go, to achieve dual-channel sales. It also runs a program called "Amazon Second Chance" to encourage customers to recycle and reuse products they no longer want. Walmart, one of the world’s largest retailers, is favored by consumers for its online and offline sales channels. And to promote sustainable development, Walmart has also launched a variety of recycling services, including waste batteries, waste pharmaceuticals, and waste electrical appliances, and encourages its suppliers to implement green production programs.

The formation of retail groups is an essential manifestation of retailer-led sustainability. This not only enhances the company’s green image but is also a favorable competitive element. On the one hand, the development of a dual-channel sales business can attract consumers with different channel preferences and expand potential market share. At the same time, the expansion of the recycling business can help brands stand out from competitors, increase brand value, build customer loyalty, and attract green consumers [10]. On the other hand, integrating a dual-channel sales operation with a recycling operation can cut fixed and variable costs by lowering transportation costs, reducing labor costs, etc., which can help companies boost profits [11]. At the same time, with the advantage of sensitivity to the market and consumers, more retail groups have become leaders in the supply chain [12–14] and have more voice in the closed-loop supply chain.

Although retail groups can bring multiple benefits, they also increase the complexity of decision-making in the supply chain system. On the one hand, consumers want both a great price and an actual product experience. This can lead to the fact that some consumers will experience the product on offline channels before purchasing the product on online channels. As a result, the offline sales channel’s service costs do not translate into benefits for itself. Instead, the online sales channel benefits. This is referred to as "free-riding" [15].

On the other hand, retail groups reinforce the inequality of power among supply chain members and raise more concerns about profit equity issues among members. Although many companies have incorporated sustainability into their future growth strategies, the primary goal for companies is still to achieve their profits [16]. When members find that their profits do not meet expectations, they often worry about whether they are being treated fair [17, 18]. For example, conflicts have arisen between Walmart and Procter & Gamble over the unfair distribution of benefits. The partnership between CVS, the largest drugstore chain in the United States, and the French pharmaceutical company Sanofi has also been affected by unfair price adjustments. Obviously, concerns about fairness will not only influence their profits but will also affect the decisions of other members of the supply chain. This is different from the traditional "rational economic man" assumption.

So, what does fairness concerns influence CLSC dominated by retail groups? Does the integration of recycling and sales operations bring changes to pricing and recycling rates in CLSC with fairness concerns? When members focus on fairness, is it conducive to the greening of CLSC?

This paper centers on the above problem by building a two-channel secondary closed-loop supply chain led by a retail group and followed by a manufacturer. On this basis, we discuss the impact of different fairness concern scenarios on closed-loop supply chain operational decisions. Unlike previous studies, the retail group in the CLSC explored in this paper is a firm with a recycling business and a multichannel sales business at the same time. Moreover, due to the influence of multi-channel sales, it is necessary to consider the existence of "free-riding" behavior between channels. We will discuss the problem of CLSC operations in three different scenarios, depending on the fairness concerns of the members. They are, Scenario 1. fairness neutrality (FN); Scenario 2, fairness concerns of the retail groups (FR); and Scenario 3, fairness concerns of the manufacturers (FM). In different scenarios, we build the corresponding models respectively. Finally, the models are solved and analyzed.

The rest of the paper is organized as follows. The next section reviews the related literature. In Section 3, we have explained the symbols and assumptions in the paper. Three models are developed in Section 4. Section 5 analyses the equilibrium solutions under different models. Finally, we summarize the main conclusions in Section 6. All proofs are provided in the S1 Appendix.

## 2. Literature review

CLSCs are a hot topic of current global concern and have received attention from a variety of sources, including companies, governments, and academia. Scholars have explored the CLSC from multiple perspectives (For example, agriculture [19, 20], transportation [21, 22], construction [23], smart manufacturing [24, 25], healthcare [26], and the platform economy [27], etc..) in different domain areas (Such as operation strategy selection [28–30], government policy influence [31–33], consumer preference [34, 35], supply chain coordination [36, 37], supply chain design [38], etc.). For the issues studied in this paper, we focus on reviewing CLSC under the domination of a retail group and CLSC issues considering fairness concerns.

### 2.1 Closed-loop supply chain

Economic globalization has brought about tremendous changes in activities such as distribution of goods, procurement, and sales, and retailers have come to prominence. While most previous studies on CLSC have taken manufacturers as leaders [31, 33, 39, 40], studies on retailers as leaders have gradually become abundant.

B. Zheng et al. [41] explored the impact of different power structures on a dual-channel CLSC and found that the rate of substitution between channels influenced the optimal power structure composition of the supply chain. Choi, Li, & Xu [42] explored the influence of different power structures of CLSC on pricing, and recovery rates. The findings indicate that the retailer-driven model leads to the greatest efficiency of the CLSC. In a three-level CLSC with recycling competition, Ranjbar et al. [43] compared consumer value-added, environmental benefits, and profit changes under a total of four scenarios: centralized scenario, manufacturer-led, retailer-led, and recycler-led. It is found that the retailer-led model is optimal for consumers, recycling rate, and overall profitability of the supply chain. Z. Zhang & Yu [44] explored the effects of different power structures in low-carbon supply chains on member pricing, emissions reduction, and recycling. It was observed that government subsidy mechanisms can significantly reduce the difference between manufacturer-led and retailer-led. Under the retailer-led situation, Wang, Y. et al. [45] analyzed the impact of altruistic preferences on decision-making in low-carbon supply chains and proposed a corresponding cost-sharing contract. When consumers have inconvenient perceptions of the recycling channel, Guo, Y. et al. [46] compare the abatement strategy choices of closed-loop supply chains under different recycling structures and find that the manufacturer-led recycling structure is optimal.

As mentioned above, scholars have also addressed the problem of CLSC decision-making under retailer domination from several perspectives, including multichannel CLSC. However, the phenomenon of free-riding between channels is also coming to the forefront due to changes in consumer behavior. In past studies, the impact of inter-channel free-riding behavior in closed-loop supply chains has been less studied, as it is more often discussed in terms of consumers’ free-riding behavior [47, 48]. There is a lack of research on the impact of inter-channel free-riding behavior in CLSC.

### 2.2 Supply chain research considering fairness concerns

Scholars have found in several behavioral experiments that fairness has an important impact on people’s daily lives and on firms’ transactional decisions [49, 50]. In a one-way supply chain, there are more abundant studies on the impact of fairness concerns, but most of the studies are manufacturer-driven [51–54].

When the retailer dominates, Pan et al. [55] explore the impact of fairness concerns on pricing for a secondary supply chain consisting of a retailer and two manufacturers. The results find that the profit of all supplier members decreases with the fair concern coefficient increase. When a strong retailer has a price squeeze and market service investment, M. Wang & Feng [56] studied the impact of manufacturer fairness concerns on supply chain performance and found that both channel performance and brand goodwill are reduced by fairness concerns.

And in CLSC, P. Ma et al. [57] examined the impact of four recycling structures on CLSC sales and recycling when retailers are concerned about fairness. X. X. Zheng et al. [2] considered a three-level CLSC decision problem with retailer fairness concerns and coordinated the supply chain using three mechanisms. Sarkar & Bhala [58] discusses recycling and coordination of CLSC when the retailer is concerned about fairness. It turns out that constant wholesale price contracts can provide effective coordination of the CLSC when the retailer’s fairness concern is large enough. When the retailer has risk aversion and fairness concerns, C. F. Li et al. [59] examine the dual-channel CLSC pricing problem. And find that the fairness concern of the retailer hurts the recycling profit and the quantity of recycled products. When having production responsibility, Liu, Z. et al. [60] explored the effect of fairness concerns on the operational decisions of CLSC and found that a win-win situation can only be created if the manufacturer cares about the retailer’s fairness concerns. All the above articles analyze the impact of fairness on CLSC decisions in a manufacturer-led context.

Although, Yao & Liu [61] introduced fairness concerns into CLSC in a retailer-led scenario. However, the study only considered retailer fairness concerns in a single-channel CLSC and did not consider a two-channel CLSC or the fairness attitudes of other supply chain members.

To this end, we attempt to explore the impact of fairness concerns on pricing and recycling in dual-channel CLSC with free-rider behavior under retailer dominance. And, considering that the strong retailer integrates the recycling business with the dual-channel sales business, we refer to it as a strong retail group for the sake of distinction.

## 3. Symbol description

This paper establishes a dual-channel CLSC consisting of a manufacturer and a retail group, where the retail group acts as the leader responsible for single-channel recycling and dual-channel sales operations, while considering inter-channel free-rider behavior. The sales business is selling products to consumers through online and offline channels. The recycling business collects and recycles used products from consumers and resells them to the manufacturer. Consumers choose online or offline channels for product purchases based on channel preference.

**Fig 1:**
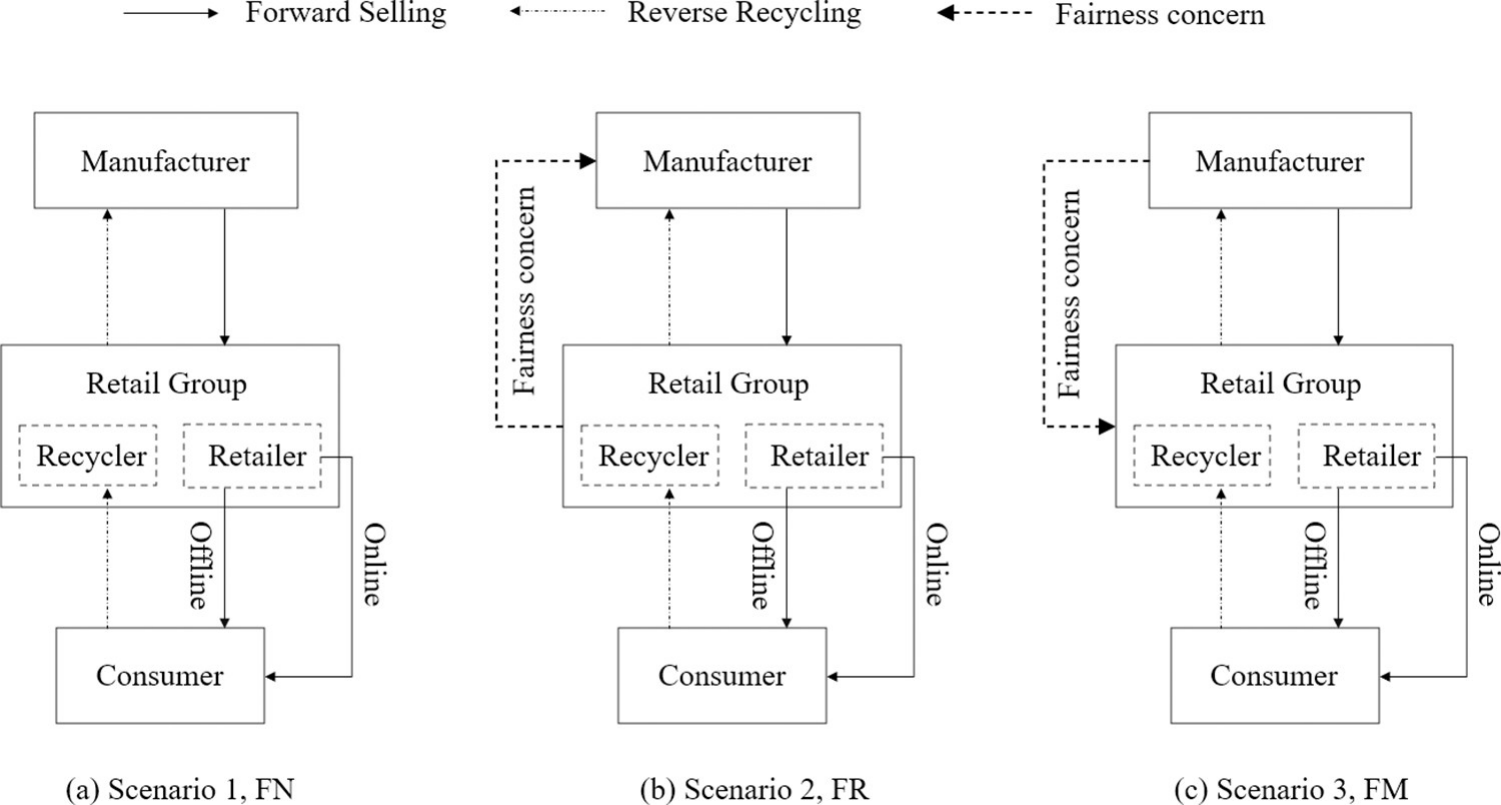
Closed-loop supply chain structure.

The manufacturer acts as a follower who is responsible for the manufacturing and remanufacturing of the product in the supply chain. On the one hand, the manufacturer needs to acquire waste products from the recycler and process the recovered waste products for remanufacturing. On the other hand, the manufacturer makes products that are wholesaled to retailers. The structure of the overall CLSC is shown in Fig 1.

Retail groups reinforce the inequality of power among supply chain members, raising more concerns about profit fairness issues among supply chain members. The three fairness concern scenarios considered in this paper are specified below: (1) Fairness neutrality (FN). Members do not feel unfair about differences in profits, and this scenario will serve as a benchmark for comparison purposes; (2) Fairness concerns of the retail groups (FR). The retail group will focus on the difference between its profits and the manufacturer and will have a fair perception of the profit difference; (3) Fairness concerns of the manufacturer (FM). The manufacturer will focus on the profit differential between itself and the retail group and will have a fair perception of the profit difference.

The relevant notations are shown in Table 1.

**Table 1:** Notations.

Symbol	Definition
*ω*	Wholesale price
*θ*	Consumers’ preference for offline sales channels, and *θ*∈(0,1)
*c*_*n*_, *c*_0_	Cost of new products, remanufacturing cost, and *c*_0_<*c*_*n*_
*p*_*d*_	Online sales price, *p*_*d*_ = *ω*+*e* [45], and *e* is marginal profit per unit
*p*_*r*_	Offline sales price, *p*_*r*_ = *ω*+*f*[45], and *f*is marginal profit per unit
*r*_*t*_, *μ*	The recovery price of the recycler, the manufacturer, and *μ*<*r*_*t*_
*q*_*r*_, *q*_*d*_	Offline sales quantity, online sales quantity
*λ*	Fairness concerns of the manufacturer, and *λ*∈(0,1)
*ξ*	Fairness concerns of the retail group, and *ξ*∈(0,1)
*τ*	The recycling rate of waste products. Cost of efforts to recover $c(\tau )=\frac{1}{2}h\tau^{2}$ [45], and *h* denotes the sensitivity factor of the recovery cost
*φ*	Manufacturer remanufacturing greenness. The green input cost is $c(\varphi )=\frac{1}{2}s\varphi^{2}$ [45], and *s* denotes the sensitivity factor for green input costs
*A*	Total sales market size
*πm*_*j*_, *πr*_*j*_ *πt*_*j*_	The profit of manufacturer, retailer, and recycler under Scenariob *j*
Π*R*_*j*_	Retail group’s profit under Scenario *j*, and Π*R*_*j*_ = *πr*_*j*_+*πt*_*j*_
*Uπm*_*j*_	Manufacturer’s utility under Scenario *j*
*U*Π*R*_*j*_	Retail group’s utility under Scenario *j*
*T*_*j*_	Total profit of the supply chain under Scenario *j*, *T*_*j*_ = *πr*_*j*_+*πt*_*j*_+*πm*_*j*_

Where, *j*∈{1,2,3} indicate respectively: Scenario 1, FN; Scenario 2, FR; Scenario 3, FM.

The development of green circular economy has enhanced consumers’ awareness of environmental protection. People will not only pay attention to the price when purchasing products, but also show concern for the greenness of the products. Mondal, C., & Giri, B. C. [62] in their study showed that more than 80% of consumers are concerned about the greenness of products. Based on their study, we set the quantity of offline channel sales for the retailer is $q_{r}=\theta A-ap_{r}+b(p_{d}-p_{r})+k\varphi$ and *a* is the sensitivity coefficient of consumers to sales price, *b* is the sensitivity coefficient of consumers to price difference between channels, *k* indicates the sensitivity coefficient of consumers to the greenness of remanufacturing, and *a*>*b*>*k*>0. Similarly, the quantity of sales in the online channel is $q_{d}=(1-\theta )A-ap_{d}+b(p_{r}-p_{d})+k\varphi$ [62].

And consumers exist offline experience online purchase behavior, offline sales channel will pay service cost *cψb*(*p*_*r*_−*p*_*d*_) for this behavior. *ψ*∈(0,1) is the proportion of free riding purchased by consumers online after offline experience [63], *c* is offline unit service cost, and *b*(*p*_*r*_−*p*_*d*_) is sales volume transferred due to the preferential online price. Each channel’s sales volume is the amount after considering any “free-rider” behavior.

## 4. Model

This section establishes three CLSC game models under different fairness concerns. They are, Scenario 1, FN; Scenario 2, FR; Scenario 3, FM. The specific game models and equilibrium solutions are as follows.

### 4.1 Scenario 1

We use this scenario as the baseline model when neither the retail group nor the manufacturer is concerned with fairness. The sales and recycling operations of the retail group are independent of each other, and the manufacturer, retailer, and recycler all aim to maximize their own profits when fairness is not considered. And according to the lead-follow relationship of the members, the decision sequence is as follows.

First, the retail group determines the online and offline sales prices. Then the manufacturer determines the wholesale price and remanufacturing greenness. The profits of the members are as follows.


$$\max(\pi r_{1})=(p_{r}-\omega )q_{r}+(p_{d}-\omega )q_{d}-c\psi b(p_{r}-p_{d})$$ (1)

$$\max(\pi t_{1})=(\mu -r_{t})(q_{r}+q_{d})\tau -\frac{1}{2}h\tau^{2}$$ (2)

$$\max(\pi m_{1})=(\omega -c_{n})(q_{r}+q_{d})+(c_{n}-c_{0}-\mu )(q_{r}+q_{d})\tau -\frac{1}{2}s\varphi^{2}$$ (3)

To facilitate the solution, the recovery rate of the recycler *τ* is assumed to be known, and then use the backward induction method to solve the model to obtain the following equilibrium solutions. The specific solution process is provided in the **Appendix A in S1 Appendix**.


$$\omega_{1}{}^{*}=\frac{As+2c_{n}(3as-4k^{2})(1-\tau )+2\tau [c_{0}(3as-4k^{2})+4\mu (as-k^{2})-asr_{t}]}{8(as-k^{2})}$$ (4)

$$\varphi_{1}{}^{*}=\frac{Ak-2ak[c_{n}(1-\tau )+\tau (c_{0}+r_{t})]}{4(as-k^{2})}$$ (5)

$$e_{1}{}^{*}=\frac{A[b+a(1-\theta )]+abc\psi }{2a(a+2b)}+\frac{c_{n}(\tau -1)+(r_{t}-c_{0}-2\mu )\tau }{2}$$ (6)

$$f_{1}{}^{*}=\frac{A(b+a\theta )-abc\psi }{2a(a+2b)}+\frac{c_{n}(\tau -1)+(r_{t}-c_{0}-2\mu )\tau }{2}$$ (7)

After verifying the Hessian matrix needs to satisfy *s*>*k*. It should be noted that to maintain their own social image, companies will guarantee their own product greenness is positive, *φ*_1_*>0. It needs to satisfy *G*>0, where $G=Ak-2ak[c_{n}(1-\tau )+\tau (c_{0}+r_{t})]$, the same in the following sections.

Bringing Eqs (4)–(7) into Eqs (1)–(3) gives the profits of the retailer, recycler, and the manufacturer, *πm*_1_*, *πr*_1_*, *πt*_1_*, respectively. And, when satisfying $h>\frac{a^{2}s(\mu -r_{t})(c_{n}-c_{0}-r_{t})}{as-k^{2}}$, there is $\frac{\partial^{2}\pi r_{1}{}^{*}}{\partial \tau^{2}}<0$. Under this condition, solve for $\frac{\partial \pi r_{1}{}^{*}}{\partial \tau }=0$ to obtain the recovery rate that maximizes the profit of the recycler.


$$\tau_{1}{}^{*}=\frac{as(A-2ac_{n})(\mu -r_{t})}{4h(as-k^{2})-4a^{2}s(\mu -r_{t})(c_{n}-c_{0}-r_{t})}$$ (8)

### 4.2 Scenario 2

This section considers when the retail group has fairness concerns. The retail group will combine the profits of both the retailer and the recycler segments, focusing on the difference in profits between itself and the manufacturer. The fairness concern utility function, $U\Pi R_{2}=(1+\xi )(\pi r_{2}+\pi t_{2})-\xi \pi m_{2}$, it indicates the utility of the retail group after focusing on the profit difference between members. The retail group will aim at maximizing utility, while the manufacturer aims at maximizing profit. Based on this, the Stackelberg game model is established and the decision order is the same as **4.1 Scenario 1**.


$$\pi r_{2}=(p_{r}-\omega )q_{r}+(p_{d}-\omega )q_{d}-c\psi b(p_{r}-p_{d})$$ (9)

$$\pi t_{2}=(\mu -r_{t})(q_{r}+q_{d})\tau -\frac{1}{2}h\tau^{2}$$ (10)

$$\max(\pi m_{2})=(\omega -c_{n})(q_{r}+q_{d})+(c_{n}-c_{0}-\mu )(q_{r}+q_{d})\tau -\frac{1}{2}s\varphi^{2}$$ (11)

$$\max U\Pi R_{2}=(1+\xi )(\pi r_{2}+\pi t_{2})-\xi \pi m_{2}$$ (12)

The solution process is the same as **4.1 Scenario 1**, and the equilibrium solutions of the supply chain are solved as follows.


$$\omega_{2}{}^{*}=\tau \mu +\frac{As(1+\xi )-2\tau asr_{t}(1-\xi )+[as(3+5\xi )-k^{2}(4+6\xi )][2c_{n}(1-\tau )+2c_{0}\tau ]}{4(as-k^{2})(2+3\xi )}$$ (13)

$$\varphi_{2}{}^{*}=\frac{Ak(1+\xi )-2ak(1+\xi )[c_{n}(1-\tau )+\tau (c_{0}+r_{t})]}{2(as-k^{2})(2+3\xi )}$$ (14)

$$\begin{array}{l} e_{2}{}^{*}=\frac{4Ab(1+2\xi )+Aa[4+7\xi -2\theta (2+3\xi )]+2abc\psi (2+3\xi )}{4a(a+2b)(2+3\xi )}\\ +\frac{c_{n}(1+2\xi )(\tau -1)+[r_{t}(1+\xi )-\mu (2+3\xi )-c_{0}(1+2\xi )]\tau }{2+3\xi } \end{array}$$ (15)

$$\begin{array}{l} f_{2}{}^{*}=\frac{4Ab(1+2\xi )+Aa[\xi +2\theta (2+3\xi )]-2abc\psi (2+3\xi )}{4a(a+2b)(2+3\xi )}\\ +\frac{c_{n}(1+2\xi )(\tau -1)+[r_{t}(1+\xi )-\mu (2+3\xi )-c_{0}(1+2\xi )]\tau }{2+3\xi } \end{array}$$ (16)

Bringing Eqs (13)–(16) into Eqs (9)–(12), we can obtain the manufacturer’s optimal profit *πm*_2_*, the retail group’s optimal utility *U*Π*R*_2_*, and the profit *πr*_2_*, *πt*_2_* corresponding to its optimal utility. When $h>\frac{2a^{2}s(\mu -r_{t})(c_{n}-c_{0}-r_{t})(1+\xi )}{\left(as-k^{2})(2+3\xi \right)}$ is satisfied, the optimal recovery rate is

$$\tau_{2}{}^{*}=\frac{as(A-2ac_{n})(\mu -r_{t})(1+\xi )}{2h(as-k^{2})(2+3\xi )-4a^{2}s(\mu -r_{t})(c_{n}-c_{0}-r_{t})(1+\xi )}$$ (17)

### 4.3 Scenario 3

This section considers the manufacturer’s concern for fairness, and the fairness concern utility function is $U\pi m_{3}=(1+\lambda )\pi m_{3}-\lambda (\pi r_{3}+\pi t_{3})$. The manufacturer will aim to maximize its utility function, and the retail group will aim to maximize profits. Based on this, the Stackelberg game model is established and the decision order is the same as **4.1 Scenario 1**.


$$\max(\pi r_{3})=(p_{r}-\omega )q_{r}+(p_{d}-\omega )q_{d}-c\psi b(p_{r}-p_{d})$$ (18)

$$\max(\pi t_{3})=(\mu -r_{t})(q_{r}+q_{d})\tau -\frac{1}{2}h\tau^{2}$$ (19)

$$\pi m_{3}=(\omega -c_{n})(q_{r}+q_{d})+(c_{n}-c_{0}-\mu )(q_{r}+q_{d})\tau -\frac{1}{2}s\varphi^{2}$$ (20)

$$\max U\pi m_{3}=(1+\lambda )\pi m_{3}-\lambda (\pi r_{3}+\pi t_{3})$$ (21)

The solution process is the same as **4.1 Scenario 1**, and the equilibrium solutions of the supply chain are solved as follows.


$$\begin{array}{l} \omega_{3}{}^{*}=\frac{A[as(1+4\lambda )-2k^{2}\lambda ]}{8a(as-k^{2})(1+2\lambda )}\\ +\frac{k^{2}\{c_{n}(2+3\lambda )(\tau -1)-\tau [c_{0}(2+3\lambda )+2\mu (1+2\lambda )-r_{t}\lambda ]\}}{2(as-k^{2})(1+2\lambda )}\\ +\frac{as\{c_{n}(3+4\lambda )(1-\tau )+\tau [c_{0}(3+4\lambda )+4\mu (1+2\lambda )-r_{t}(1+4\lambda )]\}}{4(as-k^{2})(1+2\lambda )} \end{array}$$ (22)

$$\varphi_{3}{}^{*}=\frac{Ak-2ak[c_{n}+\tau (c_{n}-c_{0}-r_{t})]}{4(as-k^{2})}$$ (23)

$$\begin{array}{l} e_{3}{}^{*}=\frac{A[a(2+3\lambda -2\theta (1-2\lambda )+2b(1+\lambda )]+2abc\psi (1+2\lambda )}{4a(a+2b)(1+2\lambda )}\\ -\frac{c_{n}(1+\lambda )(1-\tau )+[c_{0}(1+\lambda )+2\mu (1+2\lambda )-r_{t}(1+3\lambda )]\tau }{2(1+2\lambda )} \end{array}$$ (24)

$$\begin{array}{l} f_{3}{}^{*}=\frac{A[a(2\theta +4\theta \lambda -\lambda )+2b(1+\lambda )]}{4a(a+2b)(1+2\lambda )}\\ -\frac{c_{n}(1+\lambda )(1-\tau )+[c_{0}(1+\lambda )+2\mu (1+2\lambda )-r_{t}(1+3\lambda )]\tau }{2(1+2\lambda )} \end{array}$$ (25)

Bringing Eqs (22)–(25) into Eqs (18)–(21) yields the optimal profit for the retail group *πr*_3_*, *πt*_3_*, and the profit and optimal utility for the manufacturer, *πm*_3_*, *Uπm*_3_*. When satisfying $h>\frac{a^{2}s(\mu -r_{t})(c_{n}-c_{0}-r_{t})}{as-k^{2}}$, the optimal recovery is

$$\tau_{3}{}^{*}=\frac{as(A-2ac_{n})(\mu -r_{t})}{4h(as-k^{2})-4a^{2}s(\mu -r_{t})(c_{n}-c_{0}-r_{t})}$$ (26)

## 5. Analysis

### 5.1 Comparison of the three models

In this section, we analyse the equilibrium solutions for the three scenarios. For comparison purposes, we study the impact of fairness concerns on supply chain decisions when the recovery rate is known. The following propositions are obtained separately, and specific analysis process is described in **Appendix B in S1 Appendix**.

#### 5.1.1 Impact of fairness concerns on the manufacturer

Proposition 1 and Proposition 2 can be obtained by comparing the wholesale price and the greenness of remanufacturing in the three scenarios.

**Proposition 1**. Scenario 3 increases the wholesale price, while Scenario 2 decreases the wholesale price, *ω*_3_*>*ω*_1_*>*ω*_2_*, and the magnitude of the impact on the wholesale price varies across the fairness concern scenarios.

Using Scenario 1 fairness neutrality as a benchmark, it is known that in Scenario 3 (FM), the manufacturer will enhance the profit margin by increasing the wholesale price *ω*_3_* to protect the overall utility. In scenario 2 (FR), the retailer puts pressure on the manufacturer to lower the wholesale price *ω*_2_* to reduce the cost of sales and improve its overall utility.

**Proposition 2**. Scenario 2 will make the manufacturer less green, while Scenario 3 will not affect the greenness, *φ*_3_* = *φ*_1_*>*φ*_2_*. And in Scenario 2, the manufacturer greenness decreases with the increase of the fairness concern factor *ξ*, $\frac{\partial \varphi_{2}{}^{*}}{\partial \xi }<0$.

In scenario 3 manufacturer fairness concerns will first secure their utility by raising the wholesale price *ω*_3_*, rather than changing the greenness *φ*_3_*. In Scenario 2, when the retail group is concerned about fairness, the manufacturer will reduce the wholesale price *ω*_2_* and the greenness *φ*_2_* to protect its utility and respond to the retail group’s demands.

Corollary 1 can be obtained from Proposition 1 and Proposition 2.

**Corollary 1.** In scenario 2 retail group fairness concern, both wholesale price *ω*_2_* and manufacturer greenness *φ*_2_* are affected by the fairness concern coefficient *ξ* and the relationship between the rate of change is $\frac{\partial \omega_{2}{}^{*}}{\partial \xi }=\frac{s}{2k}\frac{\partial \varphi_{2}{}^{*}}{\partial \xi }$.

When the retail group is concerned about fairness, the manufacturer secures sales volume by reducing wholesale prices, while reducing greenness reduces the manufacturer’s green costs. When *s*>2*k*, the wholesale price changes faster than the greenness, $\left|\frac{\partial \omega_{2}{}^{*}}{\partial \xi }|>|\frac{\partial \varphi_{2}{}^{*}}{\partial \xi }\right|$. When 0<*s*<2*k*, the wholesale price changes at a rate smaller than the rate of change of greenness, $\left|\frac{\partial \omega_{2}{}^{*}}{\partial \xi }|<|\frac{\partial \varphi_{2}{}^{*}}{\partial \xi }\right|$.

At this point, the manufacturer will weigh the pros and cons of green costs versus benefits to protect its utility while responding to the retail group’s demands. When the green cost is higher, *s*>2*k*, the manufacturer prefers to adjust the wholesale price to respond to the supply chain members’ demands. When the green cost is low, 0<*s*<2*k*, manufacturers prefer to adjust the greenness to respond to the supply chain members’ demands.

#### 5.1.2 Impact of fairness concerns on the retail group

We analyse the equilibrium solutions for the retail group, comparing the pricing, recovery rate under each of the three fairness concern scenarios. The results obtained are illustrated in Proposition 3 and Proposition 4.

**Proposition 3.** Fairness concerns won’t affect pricing differences between online and offline, *e*_1_*−*f*_1_* = *e*_2_*−*f*_2_* = *e*_3_*−*f*_3_*. Scenario 2 retail group fairness concerns are beneficial to unit profitability across all sales channels, *e*_2_*>*e*_1_*<*e*_3_*, *f*_2_*>*f*_1_*<*f*_3_*.

Although, pricing differences between sales channels are not affected by fairness concerns, both the percentage of free-riders *ψ* and consumer channel preferences *θ* have an impact on them, $\frac{\partial (e_{j}{}^{*}-f_{j}{}^{*})}{\partial \psi }>0$, $\frac{\partial (e_{j}{}^{*}-f_{j}{}^{*})}{\partial \theta }<0$. The retailer can direct consumer ride-sharing behaviour and channel preference by adjusting unit sales margins. And, Scenario 2 increases the retailer’s profit per unit of sales and Scenario 3 decreases the retailer’s unit of sales.

Combining Proposition 1 with Proposition 3 we can clarify the change in sales price.

**Corollary 2**. The sales price of each channel is only affected by the retail group’s fairness., *p*_*d*2_*>*p*_*d*3_* = *p*_*d*1_*, *p*_*r*2_*>*p*_*r*3_* = *p*_*r*1_*.

Although, Scenario 2 will result in a lower wholesale price, it will also increase the profit per unit sold more. Ultimately, the sales price under scenario 2 is shown to be the largest. In scenario 3 manufacturer fairness concerns, although it will lead to an increase in the wholesale price, the profit per unit sold will decrease by the same magnitude. Ultimately, in scenario 3 does not affect the sales price.

**Proposition 4.** Manufacturer fairness concerns do not have an impact on the recovery rate *τ*_3_* = *τ*_1_*, while the recovery rate *τ*_2_* decreases with an increase in the fairness concern factor *ξ* when the retail group is concerned about fairness.

Fairness concerns do not promote higher recycling rate. And the more the retail group focuses on fairness, the more the recovery rate decreases. This is because decision makers are concerned with profit fairness. The retail group will not only weigh the revenues and inputs of the recycling business, but also the sales business against the recycling business. The more the retail group is concerned about fairness, the lower the recycling rate will help it reduce costs.

#### 5.1.3 Impact of fairness concerns on profits

This section compares the manufacturer profit, the retail group profit, and total supply chain system profit under different scenarios. The specific results are described in the following propositions.

**Proposition 5.** The manufacturer’s fairness concern will benefit its own profit increase, and the retail group’s fairness concern will make the manufacturer’s profit decrease, *πm*_3_*>*πm*_1_*>*πm*_2_*.

The focus on fairness is an effective way for the manufacturer to advocate for its own interests. And as the degree of equity concern increases, the greater the difference in the manufacturer’s profit between scenarios, $\frac{\partial (\pi m_{3}{}^{*}-\pi m_{1}{}^{*})}{\partial \lambda }>0$, $\frac{\partial (\pi m_{1}{}^{*}-\pi m_{2}{}^{*})}{\partial \xi }>0$. When $\lambda^{*}\geq \frac{\sqrt{(1+\xi ){\left(2+3\right)}^{3}}-2(1+\xi )}{4(1+\xi )}$, there is $\frac{\partial (\pi m_{3}{}^{*}-\pi m_{1}{}^{*})}{\partial \lambda }\leq \frac{\partial (\pi m_{1}{}^{*}-\pi m_{2}{}^{*})}{\partial \xi }$. The rate of change in the variance of the manufacturer’s profit under different scenarios is related to the fairness concerns *λ* and *ξ*.

**Proposition 6.** Fairness concerns can make the retail group less profitable, Π*R*_1_>max(Π*R*_2_,Π*R*_3_).

Specifically, Scenario 3 would not affect the profitability of the recycling business *πt*_3_*−*πt*_1_* = 0, but would make the retail business less profitable *πr*_1_*−*πr*_3_*>0. Scenario 2 would result in lower profit in the recycling business *πt*_1_*−*πt*_2_*>0 and an increase followed by a decrease in the retail business. When ξ*∈(0,*Z*), there is *πr*_1_*<*πr*_2_*. When ξ*∈[*Z*,1), there is *πr*_1_*≥*πr*_2_*.

The manufacturer’s fairness concern does not affect the recycling profit. This is because the manufacturer’s fairness concern does not affect the retailer’s selling price, so that both the number of products sold and the number of recoveries remain the same, and thus has no effect on the recovery profit. It also shows that scenario 3 does not affect the consumer’s feelings. Under scenario 2, the sales price will increase and the reduction in sales quantity will be consequently. This will affect the recovery quantity and eventually lead to a decrease in recovery profit as the retail group fairness concern increases.

**Proposition 7.** The manufacturer fairness concern does not affect the total supply chain profit, but the retail group fairness concern makes it lower, *T*_3_ = *T*_1_>*T*_2_.

The manufacturer fairness concern is the impact on profit transfer and profit distribution among members within the supply chain, without impacting the overall profit of the system. Total supply chain profit decreases as the retail group’s fairness concerns increase. At this point, there is a loss in total supply chain profit, which reflects the fact that sales prices, sales quantities, and recovery quantities are affected by the retail group’s fairness concerns, which in turn causes a reduction in overall profit.

### 5.2 Numerical analysis

In this section, the above model results are analyzed and supplemented with numerical analysis to observe more intuitively the impact of different fairness concern states and fairness concern levels on supply chain members. Referring to the studies of Jian, J., et al. [16] and Ma, P., et al. [57], the relevant values in this paper are set as shown in Table 2.

**Table 2:** Parameters setting.

Symbol	*A*	*a*	*b*	*k*	*r*_*t*_	*μ*	*c*_*n*_	*c*_0_	*θ*	*ψ*
Value	800	6	2	1	3	5	30	15	0.6	0.3

#### 5.2.1 Impact of fairness concerns on the manufacturer’s profit

**Fig 2:**
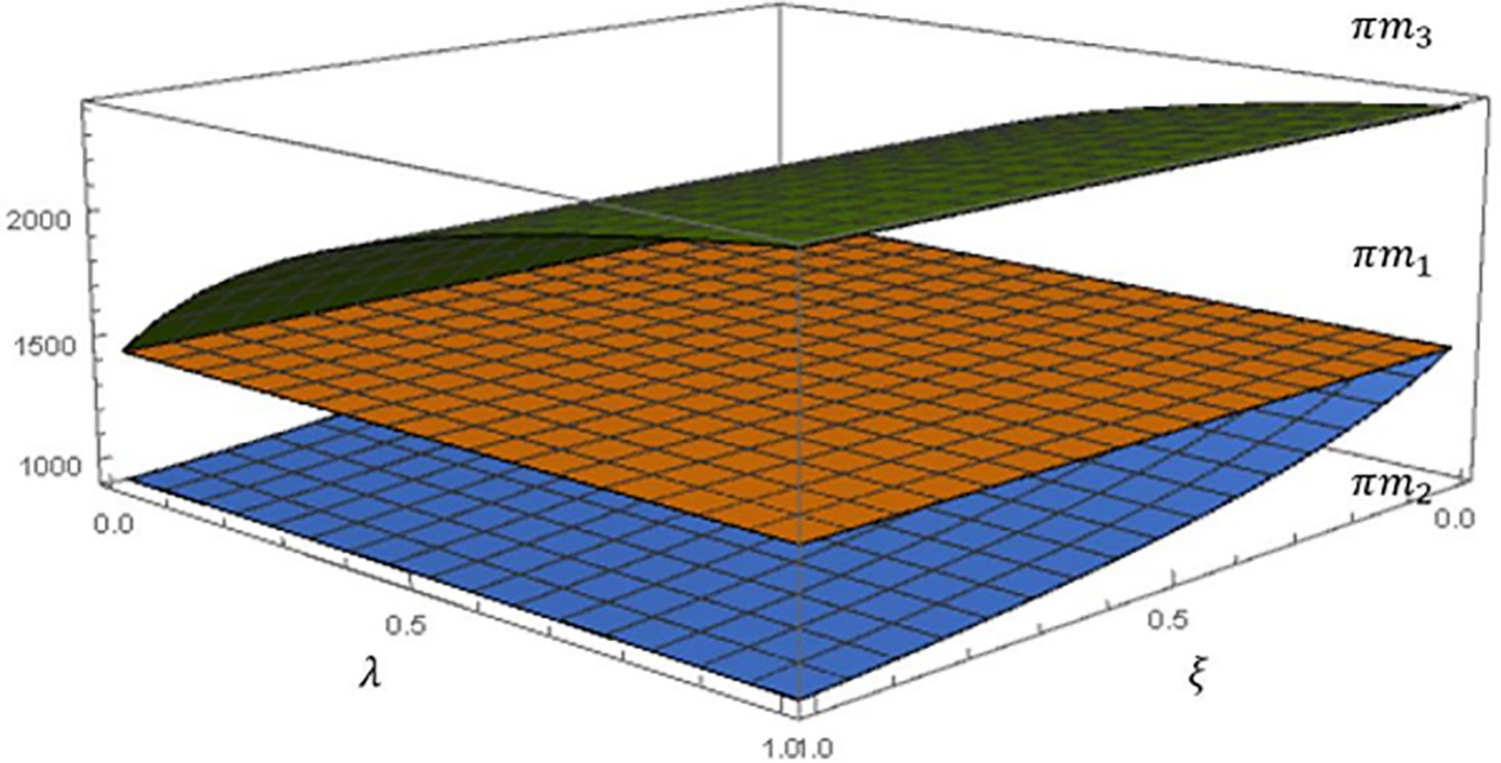
Impact of fairness concerns on the manufacturer’s profit.

To facilitate the depiction of the impact of the fairness concern scenario and the degree of fairness concern on the manufacturer’s profit, we fixed the recovery rate to *τ* = 0.6 and plotted Fig 2.

As shown above, using Scenario 1 (FN) as a baseline, we verify the conclusion in **Proposition 5**, *πm*_3_*>*πm*_1_*>*πm*_2_*. As manufacturer’s fairness concern *λ* increases, manufacturer’s profit *πm*_3_ gradually increases, but the rate of increase slows down. This is because the wholesale price increases under Scenario 3 (**Proposition 1**), while the greenness does not change (**Proposition 2**) and ultimately the overall manufacturer profit shows an upward trend. This means that the manufacturer, although a follower, can put pressure on the retail group to be responsive to its claims of fairness concerns.

In Scenario 2, as the retail group’s fairness concern *ξ* increases, the manufacturer’s profit *πm*_2_ gradually decreases and the rate of decrease slows down. This is because, Scenario 2 would allow both wholesale prices and greenness to decrease (**Proposition 1, Proposition 2**), ultimately leading to lower profits for the manufacturer. The manufacturer is willing to sacrifice its profits to satisfy the retail group’s fairness concerns to build a good partnership.

#### 5.2.2 Impact of fairness concerns on the retail group’s profit

**Fig 3:**
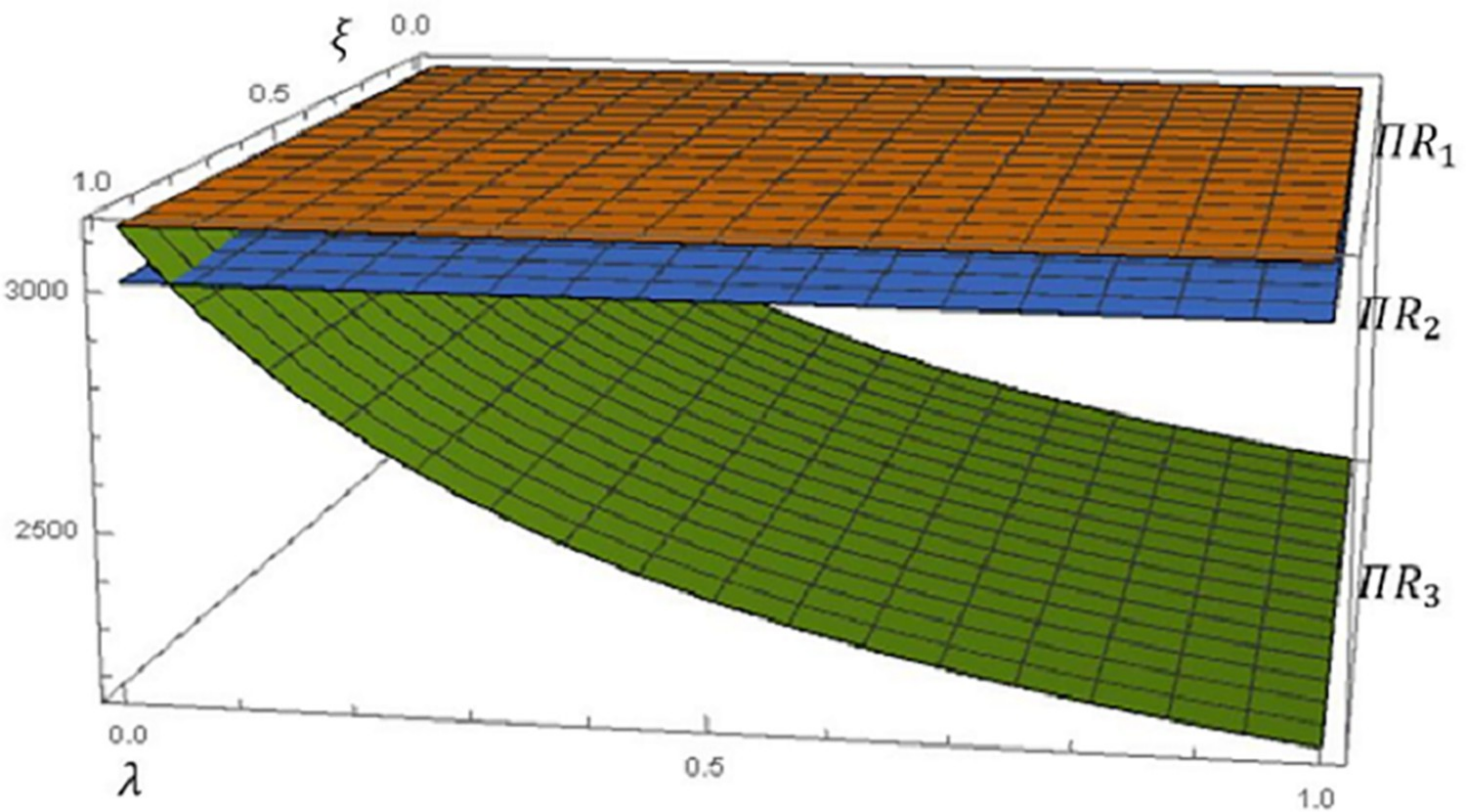
Impact of fairness concerns on retail group profits.

**Fig 4:**
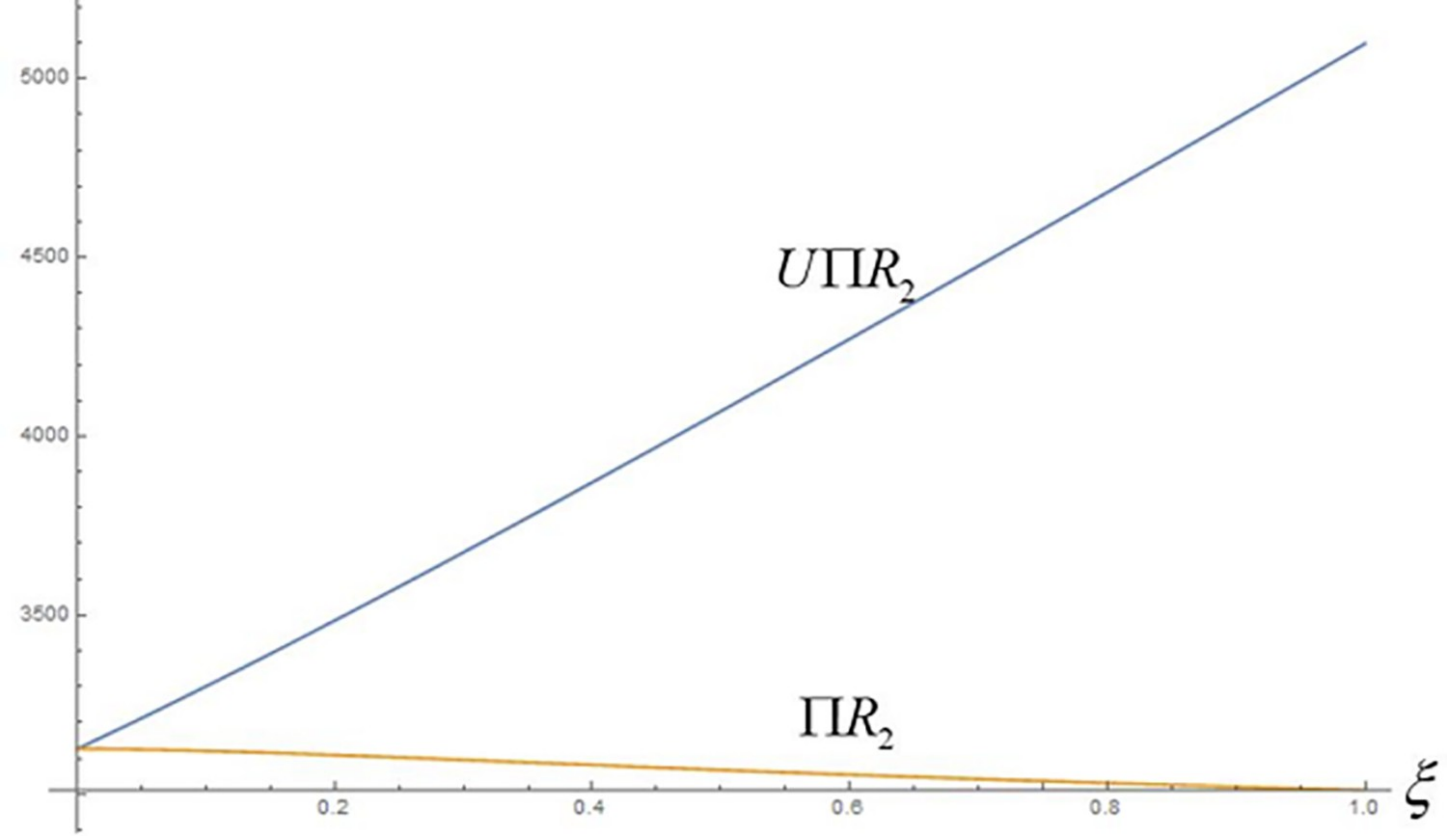
Impact of Scenario 2 on the retail group’s profit and utility.

Based on the above setup, we plot Figs 3 and 4 to depict the effect of fairness on the profitability and utility of the retail group, respectively.

Take the profit Π*R*_1_ under Scenario 1 (FN) the benchmark. From Fig 3, whenever members have fairness preference, it will reduce the retail group profit. In other words, fairness concerns are detrimental to the retail group profit (**Proposition 6**), Π*R*_1_>max(Π*R*_2_,Π*R*_3_). When a retail group focuses on fairness, on the one hand, the selling price of the product will increase (**Corollary 2**), which will affect the quantity of the product sold and lead to a decrease in the profitability of the retail business. On the other hand, the amount of recycling will decrease (**Proposition 4**) and result in lower profits for the recycling business.

Combine the multiple effects of wholesale price, sales price, and recall quantity on the retail group’s profit. When the manufacturer is less concerned about fairness, $\lambda^{**}<\frac{\xi^{2}}{4+12\xi +7\xi^{2}}$ (**Proposition 6**). The wholesale price *ω*_3_ will increase slightly, resulting in an erosion of the retailer’s profit. However, since the sales price remains stable (**Corollary 2**) and does not affect the quantity sold and the quantity recovered (**Proposition 4**), the erosion, in this case, is not significant, Π*R*_3_>Π*R*_2_. And as the manufacturer’s fairness concern increases $\lambda^{**}>\frac{\xi^{2}}{4+12\xi +7\xi^{2}}$, the wholesale price will increase gradually more. At this point, a stable number of sales and recoveries also cannot mitigate the reduced profitability of the retail group due to the large increase in wholesale price, Π*R*_3_<Π*R*_2_.

From Fig 4, it is clear that despite the negative impact of fairness concerns on the retail group’s profits, Scenario 2 has a significant increase in the total utility of the retail group, *U*Π*R*_2_>Π*R*_2_. The utility of the retail group is increased substantially with the increase in the fairness concern coefficient *ξ*. As a dominant player in the supply chain, it is crucial to maintain stable and effective operation of the supply chain. The stability and efficiency of the supply chain directly affect the competitiveness, customer satisfaction, and profit level of the enterprise. This is why the retail groups are willing to satisfy fairness concerns by sacrificing some of their profits.

#### 5.2.3 Impact of fairness concerns on the retail’s profit

**Fig 5:**
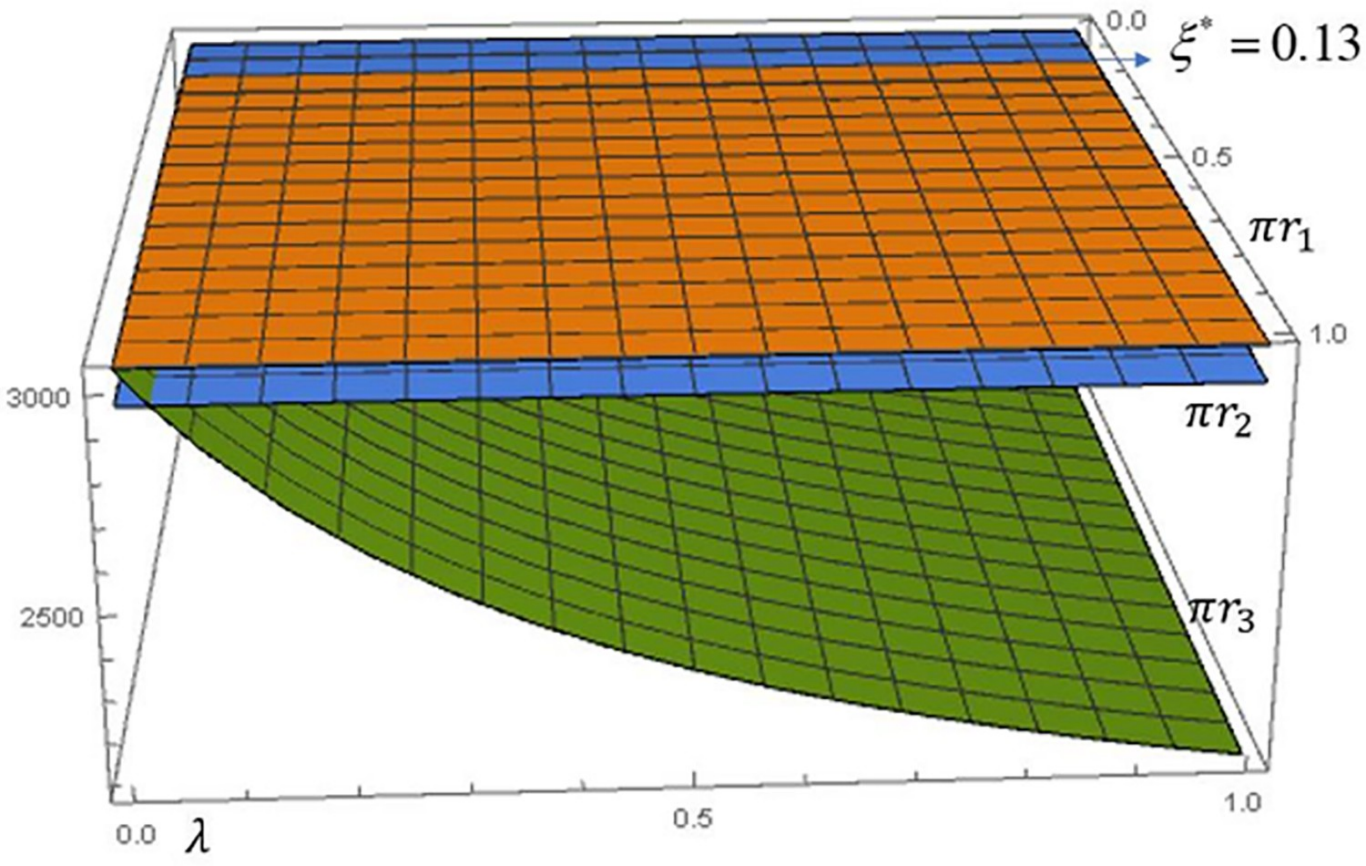
Impact of fairness concerns on the profit of the retailer.

**Fig 6:**
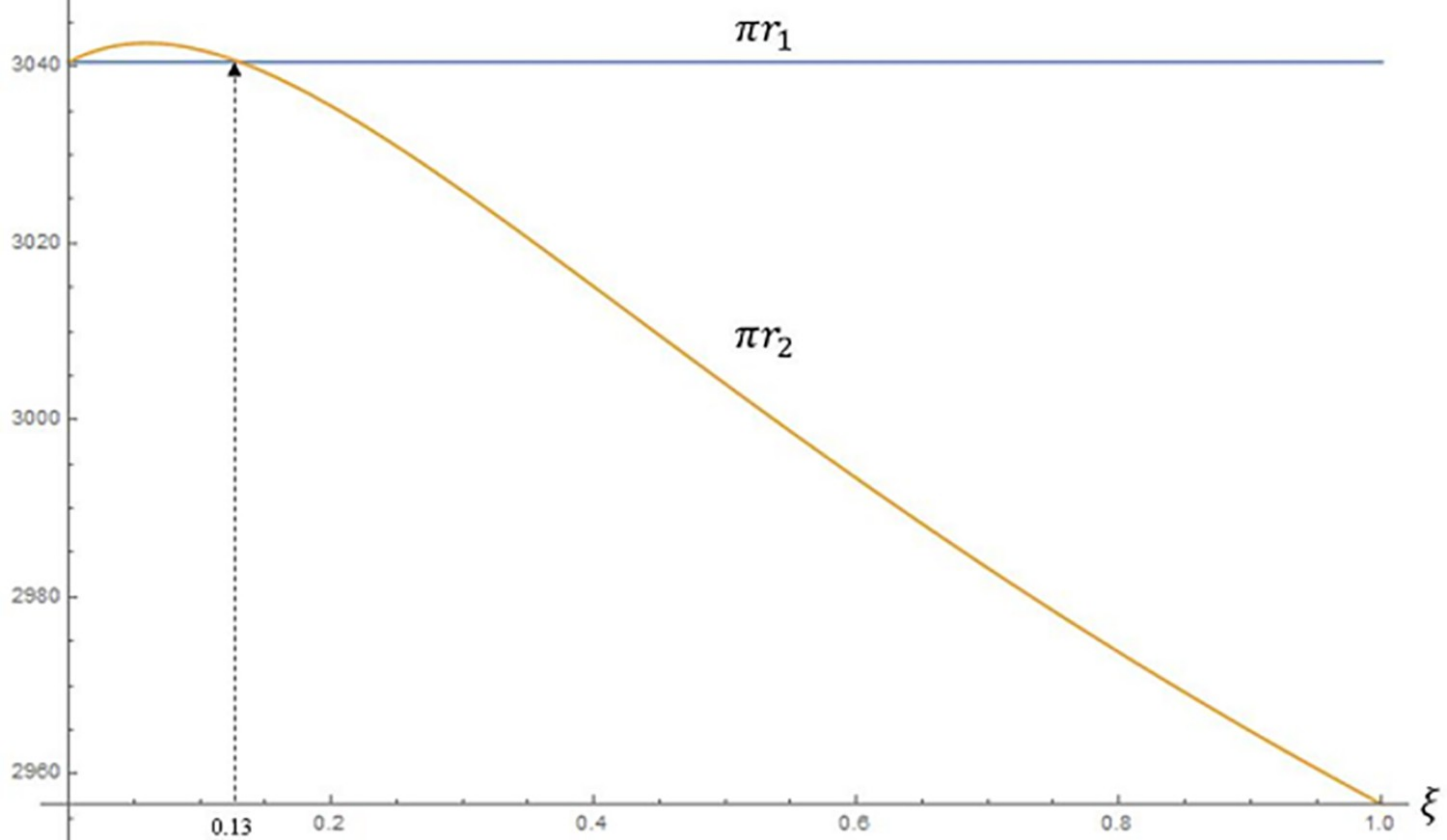
Impact of Scenario 1 vs. Scenario 2 on the profit of the retailer.

In this section, we will discuss the retail business of the retail group. Based on Table 2, we plotted Figs 5 and 6.

As shown in Fig 5, using fair neutrality as a reference, the manufacturer’s fairness concern reduces the retailer’s profit by a larger amount, *πr*_1_*−*πr*_3_*>0. This is because when the manufacturer is concerned about fairness, although the selling price remains the same, the wholesale price increases (**Proposition 1**) and the retailer’s marginal profit on sales decreases (**Proposition 3**). All these changes compress the retailer’s profit margin, resulting in a significant decline in its profit. In Scenario 2, the retailer’s profit is higher than the benchmark profit (*πr*_2_>*πr*_1_) when the fairness concern factor *ξ* is within a certain range. For perspective, we compare the retailer profits under Scenario 1 and Scenario 2 in Fig 6.

When the retail group is concerned about fairness, there is a trend of increasing and then decreasing retailer profits, as shown in Fig 6. When ξ* = 0.13, the retailer’s profit is the same in Scenario 1 and Scenario 2 (**Proposition 6**). When *ξ*∈(0, 0.13), there is a small increase in the retailer’s profit. This is because the retail group’s fairness concern leads to an increase in its marginal profit and a consequent increase in sales prices (**Corollary 2**), which leads to a decrease in sales volume. At this point, when the fairness concern *ξ* is small, the increase in marginal profit can offset the negative effect of the decrease in sales quantity, that is, *πr*_2_>*πr*_1_.

Combine Figs 5 with 6 and analyse them together. It should be noted that since the profit of the recycler decreases more than the profit of the retailer increases (*πr*_2_−*πr*_1_)<(*πt*_1_−*πt*_2_), the profit of the retail group is shown as Π*R*_1_>Π*R*_2_. When *ξ*∈(0.13,1), the increase in marginal profit has been unable to compensate for the negative effect of the reduction in sales volume, so the retailer’s profit gradually decreases.

In summary, the impact of the retail group’s fairness concern is different for the recycling business and the retail business. While Scenario 2 has a negative impact on the retail group’s profit overall, appropriate fairness concerns will bring a small boost to the profit of its retail business segment.

## 6. Concluding remarks

### 6.1 Conclusions

With the development of Internet technology and a green circular economy, more and more strong retailers are gradually opening dual-channel sales and expanding their recycling business, becoming a retail group responsible for both recycling and dual-channel sales. The inequality of power among supply chain members inevitably generates concerns about the fairness of profits. Therefore, in this paper, the impact of three different fairness concern scenarios on retail group-led dual-channel CLSC decisions is discussed. Also, inter-channel free-riding behaviour is considered. We build the game models for each of the three scenarios and solve them analytically. Taking the fair-neutral scenario as a reference, we can obtain the following conclusions.

The manufacturer’s fairness concern will increase its own wholesale prices, but the greenness of remanufacturing will not be affected. For the manufacturer, adjusting wholesale price is more likely to satisfy its own fairness claims. Both wholesale price and remanufacturing greenness decrease when the retail group is concern about fairness. The manufacturer then weighs the pros and cons of green costs versus green benefits on firm utility, and then adapts the rate of change in wholesale price and remanufacturing greenness to respond to the retail group’s fairness demands.

Although, the marginal profit difference between the two sales channels will only be affected by channel preference and free-rider ratio and will not be affected by fairness concerns. However, while the retail group fairness concern increases the marginal profit of the sales channels, the manufacturer fairness concern decreases them. In conjunction, with the effect of fairness on wholesale price, it is known that the retail group fairness concern will make the sales prices increase, which will directly affect the consumer’s purchase demand. In contrast, the manufacturer’s fairness concern has no effect on the selling prices.

The focus on fairness does not promote an increase in the recycling rate. Moreover, the more the retail group focused on fairness, the more recycling rate declined. This is because decision makers are concerned with profit fairness. The retail group will not only weigh the revenues and inputs of the recycling business, but also the sales business in relation to the recycling business. The more the retail group is concerned about fairness, the lower the recycling rate will help it reduce costs. This is where policy guidance can be used to adjust the impact of fairness concerns on the recycling rate.

The manufacturer is willing to sacrifice its own profit to respond to the retail group’s concern for fairness to build a good partnership. But no matter who focuses on fairness, the retail group profit will be lower. This is because when the manufacturer is concerned about fairness, the increase in wholesale prices still compresses the retail group’s profits, although both the number of sales and the number of recoveries are more stable. When a retail group focuses on fairness, both the number of sales and the number of recoveries are reduced, which in turn reduces the retail group’s profits. While the retail group’s fairness concerns have a negative impact on its own profits, appropriate fairness concerns can provide a small boost to the profits of the retail business unit.

The manufacturer’s fairness concern is the impact on profit transfer and profit distribution among members within the supply chain, without affecting the overall profitability of the system. The total supply chain profit decreases as the retail group’s fairness concerns increase, meaning that there is a loss in total supply chain profit. Although, fairness concerns do not contribute to the total profit of the supply chain system, they are important for the maintenance of cooperative relationships among members.

### 6.2 Managerial insights

Fairness concerns are one of the common social preferences of decision-makers when faced with unacceptable profit differentials. Especially when a retail group is formed, the expansion and dominance of the supply chain members can intensify decision-makers’ focus on profit fairness. Unlike the "rational economic man" studies, it is more relevant to study the impact of fairness concerns on closed-loop supply chain decisions after the emergence of retail groups.

The retail group’s role as a leader is key to maintaining effective long-term cooperation among supply chain members. While a focus on fairness can make a retail group less profitable, building partnerships with fairness concerns can foster better cooperation in the long run. As a decision maker, more emphasis should be placed on the advantages of a solid supply chain partnership, which not only include increased trust and loyalty, joint growth, and a favorable brand image. Also, the retail group needs to make dynamic adjustments to channel pricing, and recovery rate decisions to adapt to the changing decision-making environment. It will help the retail group to gain a longer-term competitive advantage in a highly competitive market.

The manufacturer, as a follower, making a fair claim is a practice that should be encouraged. From the results of the study, it is clear that the manufacturer’s concern for fairness does not affect the overall profitability of the supply chain system, while at the same time, it can secure for itself a certain amount of profit re-division. Maintaining a positive, cooperative attitude when making fair claims and exploring solutions to problems with partners is an effective way to realize fair claims. This not only helps to safeguard their interests and improve partner relations but also promotes the development of the industry and maintains the stable operation of the closed-loop supply chain.

The formation of retail groups increases supply chain members’ concern for profit fairness, which can adversely affect both product greenness and used product recycling rates. Governments should guide green and circular development from various aspects. For example, formulate relevant policies and regulations. Encourage supply chain members to invest in green technology and environmental protection measures. Seriously crack down on environmental violations. Educating the public on environmental protection and green products will help grow the green economy.

### 6.3 Limitation and future research direction

This study has some limitations. The article assumes that the sales and recycling operations of the retail group are independent of each other, an assumption that is very strict. Recycling activities are carried out while attracting some consumers. The retail group can use different forms of offers to attract consumers who are disposing of old products to buy new products from their group. We wanted to delve deeper into which form of offer would be more appealing to consumers selling their old products and buying new ones as well. For example, price discounts, value-added services, freebie add-ons, etc. This would be an interesting and worthwhile study, which we will discuss in a future study.

## Supporting information

S1 Appendix (DOCX)
