# Supplementary material for: A dual-channel secondary closed-loop supply chain considering retail groups and fairness concerns
Source: PLoS One. 2023 Oct 16;18(10):e0292753. doi: 10.1371/journal.pone.0292753 (PMC10578605; doi:10.1371/journal.pone.0292753)
Supplement: S1 Appendix — (DOCX) [file pone.0292753.s001.docx]

**Appendix A**

**Proof of 4.1 Scenario 1**

Step 1: Solve for the wholesale price $\omega$ and the remanufacturing greenness , and test the Hessian Matrix: (1) the first order principal subexpression is less than zero, (2) the second order principal subexpression is greater than zero.

(A.1)

The optimal wholesale price and remanufacturing greenness exist only if $-4a<0$ and $4a s-4k^{2}>0$ in the Hessian Matrix are satisfied. Combining the known condition $a>b>k>0$, it follows that the manufacturer profit function is strictly concave when $s>k$ is satisfied.

Based on the above constraints, the joint solution $\frac{\partial\pi m_{1}}{\partial\omega}=0$, , can be obtained.

$\omega_{1}\text{=}\frac{As+2c_{n}(a s-k^{2})(1-\tau)+2\tau(a s-2k^{2})(c{}_{0}+\mu)-a s (e+f)}{4(a s-k^{2})}$ (A.2)

(A.3)

Step 2: Bring (A.2) (A.3) into the retail group profit function, and similarly, determine the optimal profit per unit online and profit per unit offline. The equilibrium solution is obtained by solving for $\frac{\partial(\pi r_{1}+\pi t_{1})}{\partial f}=0$ and $\frac{\partial(\pi r_{1}+\pi t_{1})}{\partial e}=0$.

$e_{1}^{*}\text{=}\frac{A[b+a(1-\theta)]+a b c\psi}{2a(a+2b)}+\frac{c_{n}(\tau-1)+(r_{t}-c_{0}-2\mu)\tau}{2}$ (A.4)

$f_{1}^{\text{*}}\text{=}\frac{A(b+a\theta)-a b c\psi}{2a(a+2b)}+\frac{c_{n}(\tau-1)+(r_{t}-c_{0}-2\mu)\tau}{2}$ (A.5)

Step 3: Bringing (A.4) (A.5) into (A.2) (A.3), the equilibrium solution of the wholesale price and the greenness of remanufacturing is found as.

$\omega_{1}^{\text{*}}\text{=}\frac{As+2c_{n}(3a s-4k^{2})(1-\tau)+2\tau[c_{0}(3a s-4k^{2})+4\mu(a s-k^{2})-a s r_{t}]}{8(a s-k^{2})}$ (A.6) (A.7)

**Appendix B**

**Proof of Proposition 1.**

According to the equilibrium solutions, we know that $\omega_{3}^{*}-\omega_{1}^{*}=\frac{\lambda G}{4a(1+2\lambda)}>0$, and with the fairness concern factor $\lambda$ increases, the wholesale price $\omega_{3}^{*}$ increases as well,$\frac{\partial(\omega_{3}^{*}-\omega_{1}^{*})}{\partial\lambda}=\frac{\partial\omega_{3}^{*}}{\partial\lambda}=\frac{G}{4a(1+2\lambda)^{2}}>0$.

Comparing Scenario 2 with Scenario 1, it can be seen that $\omega_{2}^{*}-\omega_{1}^{*}=-\frac{s \xi G}{8(a s-k^{2})(2+3\xi)}<0$. The fairness concern factor $\xi$ increases, the wholesale price $\omega_{2}^{*}$ is gradually decreasing, $\frac{\partial(\omega_{2}^{*}-\omega_{1}^{*})}{\partial\xi}=\frac{\partial\omega_{2}^{*}}{\partial\xi}=-\frac{s G}{4(a s-k^{2})(2+3\xi)^{2}}<0$.

**Proof of Proposition 2.**

According to the equilibrium solutions, we know that . This indicates that manufacturer equity concerns do not affect optimal greenness.

Comparing Scenario 2 with Scenario 3, we know that .

The fairness concern factor $\xi$ increases, the greenness  is gradually decreasing, .

**Proof of Proposition 3.**

According to the equilibrium solutions, $e_{1}^{*}-f_{1}^{*}=e_{2}^{*}-f_{2}^{\text{*}}=e_{3}^{\text{*}}-f_{3}^{\text{*}}=\frac{A(1-2\theta)+2bc\psi}{2a\text{+4}b}$. This suggests that different fairness scenarios do not affect pricing differences between sales channels.

According to $\frac{\partial e_{j}^{*}-f_{j}^{*}}{\partial\psi}=\frac{bc}{a+2b}>0$, it can be known that as the percentage of free-riders increases, the price difference between sales channels increases. And when consumers' offline channel preference increases, pricing differences between sales channels decrease, $\frac{\partial e_{j}^{*}-f_{j}^{*}}{\partial\theta}=\frac{-A}{a+2b}<0$.

**Proof of Proposition 5.**

Using Scenario 1 as a baseline, all other different scenarios have the manufacturer's profit variance increase as the level of fairness concern increases. More specifically, $\frac{\partial\pi m_{3}^{*}-\pi m_{1}^{*}}{\partial\lambda}=\frac{s G^{2}}{16(a s-k^{2})(1+2\lambda)^{2}}>0$, $\frac{\partial\pi m_{1}^{*}-\pi m_{2}^{*}}{\partial\xi}=\frac{s(1+\xi) G^{2}}{4(a s-k^{2})(2+3\xi)^{3}}>0$.

**Proof of Proposition 6.**

The size of the retail group profits is compared for the three scenarios, $\Pi R_{1}>(\Pi R_{2},\Pi R_{3})$. A comparison of profits under scenario 2 and scenario 3 shows that $\Pi R_{3}-\Pi R_{2}=\frac{s[\lambda(4+12\xi+7\xi^{2})-\xi^{2}][A-2a(c_{n}-c_{n}\tau+c_{0}\tau+r_{t}\tau)]^{2}}{16(k^{2}-a s)(1+2\lambda)(2+3\xi)^{2}}=0$. When $\lambda^{\text{**}}\geq\frac{\xi^{2}}{4+12\xi+7\xi^{2}}$, there is $\Pi R_{3}-\Pi R_{2}<0$. When $\lambda^{\text{**}}<\frac{\xi^{2}}{4+12\xi+7\xi^{2}}$, there is $\Pi R_{3}-\Pi R_{2}>0$.

Comparing the profit of the retail business shows that $\xi^{\text{*}}\in(0,Z)$, $\pi r_{1}^{\text{*}}<\pi r_{2}^{\text{*}}$. When $\xi^{\text{*}}\in\left[Z \text{，} 1\right)$, there is $\pi r_{1}^{\text{*}}\geq\pi r_{2}^{\text{*}}$. And, $Z=\frac{8a\tau(\mu-r_{t})}{A-2a[c_{n}(1-\tau)+\tau(c_{0}+6\mu-5r_{t})]}$.
